# HER2+ Breast Cancer Escalation and De-Escalation Trial Design: Potential Role of Intrinsic Subtyping

**DOI:** 10.3390/cancers14030512

**Published:** 2022-01-20

**Authors:** Coralia Bueno Muiño, Miguel Martín, María del Monte-Millán, José Ángel García-Saénz, Sara López-Tarruella

**Affiliations:** 1 Medical Oncology Department, Hospital Infanta Cristina (Parla), Fundación de Investigación Biomédica del H.U. Puerta de Hierro, Majadahonda, 28009 Madrid, Spain; coralia.bueno@salud.madrid.org; 2 Medical Oncology Department, Hospital General Universitario Gregorio Marañón, Instituto de Investigacion Sanitaria Gregorio Marañon (IiSGM), CIBERONC, Geicam, Universidad Complutense, 28007 Madrid, Spain; mmartin@geicam.org; 3 Medical Oncology Department, Hospital General Universitario Gregorio Marañón, Instituto de Investigacion Sanitaria Gregorio Marañon (IiSGM) CIBERONC, 28007 Madrid, Spain; maria.delmonte.externo@salud.madrid.org; 4 Medical Oncology Department, Hospital Clínico San Carlos, Instituto de Investigación Sanitaria San Carlos (IdISSC), CIBERONC, 28040 Madrid, Spain; jgsaenz@salud.madrid.org

**Keywords:** early, HER2+, escalation, de-escalation, intrinsic subtype, HER2-enriched

## Abstract

**Simple Summary:**

Classical clinical research has been developed according to immunohistochemical breast cancer subtypes, instead of designing trials specifically for each molecular subtype. Efforts in de-escalating treatment should focus on identifying a subgroup of HER2 oncogene addicted tumours that are especially sensitive to anti-HER2 therapies and, thus, spare unnecessary treatments. A prognostic assay that integrates molecular tumour features with clinical and pathologic variables and accurately defines a group of HER2 addicted tumours remains the best candidate among these strategies.

**Abstract:**

Long-term outcomes in breast cancer patients differ based on the molecular subtype, with HER2-E being the most aggressive one. Advances in clinical practice have dramatically shifted HER2+ breast cancer prognosis. Risk adapted strategies to individualize therapies are necessary. De-escalation approaches have been encouraged based on the risks of clinical-pathological factors. Molecular gene subtyping could further accurately define HER2 addicted tumours that are sensitive to anti-HER2 therapies, thus sparing unnecessary treatments. The transition from immunochemistry to molecular profiling in HER2+ breast cancer is discussed.

## 1. Introduction

Four intrinsic subtypes of breast cancer (BC) were identified from gene expression data in 2000: Luminal A (LumA), Luminal B (LumB), HER2-enriched (HER2-E) and Basal-like BC. These subtypes have different outcomes and variable responses to anticancer therapies. The prognosis of each subtype depends on the hormone receptor (HR) status, proliferation as per Ki-67 and HER2 protein expression. Approximately 16–18% of all BC cases are HER2+ according to immunochemistry (IHC) protein expression profile [1]. A 50-gene signature (PAM50) was later developed to simplify the former subtype classifications through quantitative real-time reverse-transcription PCR (qRT-PCR) and NanoString nCounter^®^ [2]. PAM50 is currently successfully used in multiplexed gene expression platforms such as NanoString nCounter^®^, which is the basis for the Prosigna^®^ test. This test has been approved in current practice as a prognostic and predictive tool in HER2- and positive estrogen receptor (ER+) early BC and it acts as a powerful assay for clinical trial drug development [3]. To add complexity, all these intrinsic molecular subtypes can be identified in HER2+ tumours, with the HER2-E being the most frequent (~47%), followed by Luminal B (~18–28%), Luminal A (11–23%) and Basal-like (7–14%). Furthermore, this distribution seems to be heavily influenced by HR status, with HER2-E subtype representing 30% of the molecular subtypes within HR+/clinically HER2+ BC and 75% within HR-/clinically HER2+ tumours. Of note, the concordance rate between the pathology-based subtypes and the intrinsic subtypes is moderate (67.4%; kappa statistic: 0.50) [4,5]. Moreover, there is enough clinical evidence to support the idea that intrinsic subtypes might identify a subgroup of clinically HER2+ tumours that highly respond to anti-HER2 therapies regardless of chemotherapy and HR status [6]. Despite the improvement in the molecular subtyping of BC, most treatment strategies still rely on the classical pathological assessment using IHC. However, recent studies suggest that IHC is not a reliable surrogate of genomic intrinsic subtypes, and that gene expression methods have a higher predictive and prognostic value [7,8]. This argument raises concern about whether the current IHC-based classification neglects real tumour biology, thus leading to untailored treatments [9]. In fact, classical clinical research has been developed according to IHC BC subtypes, consigning the impact of intrinsic subtypes to exploratory analysis, instead of designing trials specifically for each molecular subtype. Therefore, it is currently the primetime for molecular based clinical trial designs.

PAM50 may be performed on both digital multiplexed gene expression and RNA-Seq platforms. The NanoString nCounter^®^ system enables gene expression analysis through direct multiplexed measurements. It was proven to be highly reproducible and has shown consistency between fresh-frozen (FF) and formalin-fixed paraffin-embedded (FFPE) derived RNAs. Therefore, it is a suitable tool to be used in clinical practice. On the other hand, full RNA-Seq is a fundamental research tool for whole transcriptome analysis. However, being very expensive and time-consuming renders it difficult to be used in routine clinical practice. Fortunately, limitations on removing partially degraded mRNA in FFPE samples with typical RNA-Seq protocols may be overcome by using Ribo-Zero-Seq [10].

The intriguing question is whether the data of intrinsic subtypes from different clinical trials can be compared with each other, considering that the molecular assessment has not been performed with a uniform method within all these studies. As an example, the distribution of intrinsic subtypes in neoadjuvant trials differs among similarly based IHC cohorts when using different technologies. While PAMELA and APT trials reported a similar distribution of HER2-E subtype when using the nCounter platform (66.9% and 65%, respectively), this percentage was quite different in the CALGB40601 trial, which used RNAseq (31%) [11].

This issue may be resolved by comparing the distribution of subtypes using both platforms within the same rather than different series. Consequently, some research groups have explored the correlation between gene expression data according to these two technologies [12]. Picornell et al., compared the PAM50 intrinsic subtype determination results with both assays in the same triple negative IHC-based BC series and reported a convincing 96% positive correlation [3]. Furthermore, a combined analysis of CALGB 40601 (Alliance) and PAMELA trials using RNAseq showed a high consistency with the nCounter platform. A similar rate of HER2-E (62%) was found in this combined cohort [13].

An emerging paradigm for safe clinical development aims to identify patients at risk of relapse that deserve salvage treatments or, conversely, to select patients that can safely spare the intake of unnecessary drugs. The challenging issue is the accurate stratification of these categories. Based on this, Prat et al., developed a prognostic assay that integrated molecular tumour features with clinical and pathologic variables in patients with newly HER2+ BC from the adjuvant Short-HER trial. The final prognostic model was evaluated in an independent combined dataset of patients from four neo/adjuvant studies. HER2DX score was significantly associated with metastasis-free survival as a continuous variable but the HER2DX score was not associated with pCR likelihood. [14]. To solve this issue, the same author developed and validated an additional HER2DX pCR score using clinical variables (tumour size, nodal staging) and 4 gene expression signatures (tracking immune infiltration, tumour cell proliferation, luminal differentiation, and the expression). Whereas some HER2DX variables were associated with pCR (i.e., immune, proliferation and HER2 amplicon), others were associated with non-pCR (i.e., luminal, and tumour and nodal staging). The authors concluded that both HER2DX tests (HER2Dx Risk Score and HER2Dx pCR likelihood score) provide accurate estimates of the risk of recurrence (HER2Dx Risk Score) and the likelihood to achieve a pCR (HER2Dx pCR score) in the early stage HER2+ BC [15]. Therefore, these tools might select candidates for escalated or de-escalated systemic treatment.

## 2. Anti-HER2 Clinical Development in Early BC

In the absence of specific therapy, HER2+ BC is associated with more aggressive behaviour in terms of recurrence rate and mortality, when compared to HER2 -non-overexpressed tumours. However, the emergence of highly effective HER2-targeting drugs has dramatically reversed the current management of this disease. Despite this sustained impact of HER2-targeting therapies, nearly 1 out of 4 early HER2 BC patients experience relapse, emphasizing the importance of biology over any other variable [16,17,18]. A better understanding of the molecular tumour biology, HER2 pathway and the mechanism of resistance to trastuzumab has enabled the development of novel drugs such as monoclonal antibodies, small molecule tyrosine kinase inhibitors and antibody-drug conjugates that may overcome this resistance. Paradoxically, salvage approaches do not guarantee better long-term outcomes. Therefore, adopting a “one size fits all” strategy lacks rationale. Instead, deeper insights into molecular subtypes may allow for the identification of treatment benefits across these risky subtypes. Consequently, therapy could be tailored for each specific molecular subtype. Escalated strategies have been promoted considering the risk of recurrence according to clinical-pathological factors. Noteworthy, some of these anti-HER2 drugs have led to clinically significant outcomes, while the incremental benefits of some others have been modest [19]. In the adjuvant setting, adding trastuzumab to chemotherapy halved the recurrence and mortality risk, compared with chemotherapy alone [20]. At the other end of the spectrum, the addition of pertuzumab in the adjuvant setting resulted in a marginal improvement in invasive DFS in the intention-to-treat population [21].

Hence, there is still a long road ahead as we are still not able to clearly distinguish which patients are at risk. Whereas more than half of the patients treated with chemotherapy alone in the large adjuvant trastuzumab trials were recurrence-free at 10 years, more than 10% of patients experienced an invasive event after receiving multiple anti-HER2 drugs and chemotherapy in the KATHERINE trial [22]. This highlights the importance of heterogeneity, not only of tumour biology, but also in response to treatments. Molecular profiling might solve this query as HER2+ BC is a biologically heterogeneous disease, and all four intrinsic molecular subtypes can also be identified in this population. Therefore, advances in molecular subtyping through gene signatures deserve new clinical trial designs targeting specific populations. (Table 1) [11,13,23,24,25,26,27].

## 3. Post-Neoadjuvant Guided Therapy

Standard treatment of early HER2+ BC has shifted from the adjuvant to the neoadjuvant setting. Pathological complete response (pCR) following neoadjuvant treatment correlates with good long-term survival outcomes [30]. Thus, considering that trastuzumab and/or pertuzumab combined with taxane-based chemotherapy provides pCR rates as high as 60%, and reaches 75% in HER2+/ER- tumours, this regimen stands as the neoadjuvant standard therapy [31]. Completion of 1 year of adjuvant trastuzumab was an accepted practice, irrespective of the pathological response. However, single-agent adjuvant T-DM1 demonstrated improved outcomes in patients with residual disease after neoadjuvant chemotherapy with single or dual HER2 blockade. Thus, this strategy has become the standard treatment strategy.

This approach represents a first step for risk-adapted clinical trial design. In fact, the challenge of this novel design lies on identifying higher versus lower risk tumours and then matching the drug to the individual patient. Escalation strategies in high-risk patients and de-escalation strategies in low-risk patients have been classically based on clinic-pathological issues. However, with this criterion, it is likely to over-treat a substantial number of patients or even offer disparity options to the same patient. As an example of this paradoxical scenario, a 2 cm size tumour without nodal involvement can be treated, either with upfront surgery followed by mono-chemotherapy plus trastuzumab, or with neoadjuvant poly-chemotherapy plus dual HER2 blockade. The challenge in that case is that more is not necessarily the best, and at the same time, less must not be too little. As a result, the “one size fits all” strategy for cancer treatment must be ceased. Instead of classical variables, therapy must be tailored to fit a specific molecular subtype. Perhaps, the key for this risk-adapted strategy relies, not only on estimating the overall risk of recurrence but on selecting high risk patients according to their biological driver. Therefore, de-escalating molecular stratified strategies may avoid unnecessary toxicities without compromising outcomes [32,33].

HER2-E subtype, according to PAM50, represents 60% of HER2 BC types and it is associated with a higher probability of achieving a pCR than non-HER2-E disease following neoadjuvant plus anti-HER2 therapy. This higher probability of pCR rate is mostly related to the contribution of the ER-/HER2+ cohort, as there is a strong correlation between pCR and lack of ER expression. A recently published meta-analysis including 2190 patients from prospective trials concluded that the HER2-E subtype predicts pCR for HER2 targeted neoadjuvant therapies (single HER2 targeted therapy OR: 3.36, 95% CI: 2.25–5.02 *p* < 0.001; dual HER2 targeted therapy OR: 4.66, 95% CI 3.56–6.10, *p* < 0.002) [34]. HER2-E subtype is characterized by higher expression levels of ERBB2, ERBB2-amplicon genes (eg, GRB7), and receptor tyrosine kinases, including FGFR4 and EGFR, and lower expression of luminal-related genes compared to the luminal subtypes. Moreover, HER2-E subtype has the highest activation of the EGFR–HER2 pathway [35]. This subtype is predicted to respond well to HER2 therapies. Indeed, in four neoadjuvant trials, pCR rates were higher (sometimes doubled) in the HER2-E subtype than other luminal subtypes, regardless of the treatment regimen [26]. This data suggests that HER2+/HER2-E tumours benefit the most from chemotherapy plus anti-HER2 therapy. Hence, HER2 status is clearly not enough to select patients who benefit the most from these therapeutic agents. Molecular profiling could accurately define this predictive value. So, the main concern in this HER2-E population is whether the de-escalating strategy should be focused on eliminating chemotherapy when double HER2 blockade is planned, or should it be focused on double HER2 blockade (to single) when maintaining poly-chemotherapy.

## 4. De-Escalating Strategies and Molecular Subtype Based De-Escalating Strategies

There have been substantial efforts to identify genomic predictors of pCR to guide treatment de-escalation in the neoadjuvant setting.

### 4.1. Chemotherapy Free Anti-HER2 Treatment

As neo/adjuvant lapatinib in combination with trastuzumab had not shown any event-free survival benefit in individual trials, it was considered to have no role in this context. However, a recent meta-analysis of four phase II-III trials testing lapatinib in combination with neoadjuvant trastuzumab plus chemotherapy for HER2+ early BC has provided robust evidence that this dual HER2 blockade prolongs overall survival (HR 0.65, 95% CI 0.43–0.98). This suggests that lapatinib could be repurposed in early settings. This benefit was especially relevant in hormone receptor (HR) negative tumours [36]. Noteworthy, TBCRC006/023, PER-ELISA and SOLTI-PAMELA studies revealed a 30% pCR rate with trastuzumab and lapatinib without chemotherapy [35]. The pCR rates of intrinsic subtypes were provided in the PAMELA trial showing 41% in the breast in HER2-E subtype following dual HER2 blockade [23]. Though this rate is far from what current salvage therapy may provide, it is appealing and deserves a larger prospective validation.

Furthermore, in the NeoSphere trial, adding Pertuzumab to a regimen based on trastuzumab and taxane improved pCR rate in the neoadjuvant setting, suggesting better progression-free survival. In the same study, a remarkable 16.8% pCR rate was obtained through a chemotherapy-free dual anti-HER2 regimen [37]. These data suggests that a subgroup of patients with early stage HER2+ tumours might safely be spared chemotherapy. However, beyond hormone receptor status, no predictive biomarker of response to HER2-blockade has shown clinical utility. Therefore, additional biomarkers are required to stratify risks for patients and to optimize treatment selection.

Molecular profiling may become a useful tool to select patients who can spare chemotherapy. A retrospective mRNA-based analysis for ERBB2, using qRT-PCR for baseline tumour samples from the chemotherapy-free arm, showed 23.4% and 10.9% pCR rates in ERBB2-high and ERBB2-low groups, respectively [38]. Thus, both HER2-E subtypes and the high level of ERBB2 may provide additional information that can aid in the choice of the optimum therapeutic strategy. Tumours that have a high expression signature of the main drug target and excessive activation of the HER2 and/or EGFR pathway (i.e., ERBB2-high and HER2-E) are potentially most sensitive to anti-HER2-targeted therapies. This generated the hypothesis that HER2 oncogene-addicted tumours could respond to treatment without the need for chemotherapy. Based on this, Prat et al. tested an RNA-based assay that combines ERBB2 and the HER2-E intrinsic subtype in HER2+ patients treated with dual HER2-blockade (mostly lapatinib/trastuzumab) without chemotherapy in five neoadjuvant and one additional advanced trial. Overall, HER2-E was a consistent biomarker of pCR following this dual blockade and the mRNA levels of both HER2-E and ERBB2 provided additional information either individually or when combined into a single variable; the combined variable provided better results. The study concluded that this assay could aid in the de-escalation of chemotherapy in approximately 40% of patients who are highly responsive to HER2-targeted therapy [35]. As a result, these authors introduced the novel concept of HER2 addicted tumour [39].

In the same manner, another group constructed a multiparameter classification approach that is able to predict pCR with targeted therapy alone, compared to pCR rates of chemotherapy plus dual anti-HER2 in unselected patients. This classification approach included the gene and protein levels of HER2, intra-tumour heterogeneity (ITH), HER2-E subtype, and the mutation status of PIK3CA. This approach could also identify tumours “addicted” to HER2 signalling. However, it is necessary to prospectively validate these findings in a clinical trial [40].

Both HER2-E subtypes and ERBB2 levels aim to translate the magnitude of dependence of the HER2 pathway, although they are not fully consistent. The HER2-E profile focuses on the level of activation of the HER2 signalling pathway, while ERBB2 mRNA levels ultimately determine the number of targets present in the tumour cells. Tumours that harbour both HER2-E and ERBB2-high are classified as “HER2-addicted” tumours and, therefore, are considered the most sensitive tumours to anti-HER2-targeted therapies. Conversely, HER2 sensitivity of tumours that meet only one criterion is similar to non-HER2-E/ERBB2-low disease. This could explain why not all HER2+ tumours respond in the same manner to HER2-targeted therapies. Nevertheless, pCR rates upon double HER2 blockade without chemotherapy are below the pCR rates of salvage therapies, even in HER2 addicted tumours. Perhaps a different approach should be employed such that chemotherapy is retained and combined with a single HER2 blockade in these extremely HER2 addicted tumours [35].

Similarly, as multigene assays estimate the risk of recurrence and benefit from chemotherapy in HER2 negative BC, a platform that includes molecular features, such as extent of HER2 addiction, the genomic and molecular makeup of the tumour, intra-tumour heterogeneity, and the immune milieu is required [41]. Whether such a molecular classification approach combined with conventional clinicopathological characteristics will help to further refine patient stratification remains to be explored in prospective clinical trials.

The attempt of de-escalating chemotherapy has also been explored in tumours co-expressing (HR) and HER2, benefiting from what hormonal therapy may provide. However, data must be interpreted with caution. The behaviour of HR+/HER2+ tumours differ from HR-/HER2+ tumours, with significantly lower pCR rates, less association of pCR with outcomes and smaller benefit differences with augmented anti-HER2 therapy [42]. Intrinsic subtype distribution also differs significantly according to the HR status, with a greater percentage of HER2-E in HR-negative patients [29]. However, HER2-E tumours remain sensitive to anti-HER2 therapy. For instance, pCR rate was significantly higher in HER2-E than in other subtypes (45.5% vs. 13.8%, *p* = 0.042) in a phase II PerELISA trial in HR+/HER2 BC patients receiving neoadjuvant pertuzumab trastuzumab and letrozol [27].

### 4.2. De-Escalating Chemotherapy Drugs: Anthracyclines

Considering the number of targeted therapies available for HER2+ BC to date, it is difficult to justify the use of anthracyclines. Although the rate of symptomatic heart failure with the use of anthracycline-based/trastuzumab-based therapy is low, a small but significant group of those patients were unable to complete full course of trastuzumab due to cardiac-related adverse effects. Conversely, treatment-related cardiomyopathy with a regimen sparing anthracycline, such as docetaxel-carboplatin-trastuzumab (TCH) regimen, is remarkable [43,44]. Considering that both approaches result in similar pCR rates and disease-free survival is achieved in both cases, an anthracycline-free regimen can be safely considered as the standard neo/adjuvant regimen [19].

To support this argument, TRAIN-2 trial showed no significant differences, either in pCR rate or in EFS, when anthracyclines were added to a carboplatin-taxane regimen when given in combination with the HER2-targeted agents, trastuzumab and pertuzumab; however, higher cardiotoxicity was observed in the group receiving anthracyclines. Therefore, excluding anthracyclines might be a preferred approach in the presence of dual HER2 blockade [45,46]. There is a lack of data related to the selection criteria of patients who could benefit from anthracyclines according to the intrinsic subtypes of the tumour.

### 4.3. De-Escalating Chemotherapy Drugs: Taxanes

Different studies have attempted to assess whether taxanes can be excluded or replaced to reduce their related toxicity.

The WSG-ADAPT phase II trial evaluated the efficacy of a 12-week treatment with neoadjuvant trastuzumab, pertuzumab, with or without weekly paclitaxel in HER2+/HR- patients. An outstanding pCR rate of 90.5% was achieved with chemotherapy plus dual blockade compared to the pCR rate of 36.3% observed in the free chemotherapy arm, which is comparable to pCR rates reported with more aggressive regimens [47].

These data suggest that chemotherapy plays a role, but better tolerated regimens can generate the same favourable outcomes as more aggressive schemes. Data related to intrinsic subtypes would consolidate this concept.

Conversely, since T-DM1 showed no inferior efficacy, but better tolerability than taxane plus trastuzumab in the first-line setting, different studies explored the role of T-DM1 +/- pertuzumab in the neo/adjuvant scenario.

PREDIX HER2 trial demonstrated similar efficacy and less toxicity favouring neoadjuvant TDM-1 monotherapy when compared to pertuzumab, trastuzumab, and docetaxel, particularly in patients with HER2+/HR+ cancers [48].

The KRISTINE study failed to show a higher pCR rate when T-DM1-pertuzumab was compared to TCHP in the neoadjuvant setting. Although this trial did not meet the primary endpoint, it provided remarkable data regarding molecular subtypes. HER2-E was the most common intrinsic subtype (54.8%) in ER-/HER2+ tumours and was associated with the highest pCR rate with both regimens: 72% (TCHP) and 62% (TDM-1/P). Of note, a notable 70% pCR rate was achieved in basal-like subtype in TCHP arm, suggesting the remarkable benefit of carboplatin in this subtype [49].

KAITLIN was an adjuvant trial comparing the same combination (T-DM1-pertuzumab) to taxane-trastuzumab-pertuzumab (THP) after anthracycline-based therapy in a very high-risk population. Individual biomarkers were analysed and reported remarkable results, though intrinsic subtype analysis has not been carried out. AC-TDM-1-Pertuzumab did not reduce the risk of an invasive disease free survival (iDFS) event in any of the subpopulations defined according to the biomarkers’ status, including HER2 expression, PIK3CA mutations, and others. Additionally, no prognostic relationships were observed in cohorts with higher HER2 mRNA or protein expression. No biomarker beyond HER2 showed clinical utility in HER2+ BC [50,51]. This analysis suggests that the predictive value of a single biomarker lacks clinical relevance as a universal variable in all HER2 patients. A more comprehensive molecular tool is needed to identify an individualized strategy and the molecular PAM50 subtyping may be fundamental to solve these issues.

However, not all uncertainties related to anticancer drug efficacy may be deciphered with the intrinsic subtypes. Intratumoral heterogeneity may stand the cornerstone of anti-HER2 therapy. In terms of HER2 expression levels, several studies (i.e., MARIANNE, EMILIA, TDM4450) have explored whether HER2 expression levels can predict the benefit from T-DM1 [52,53,54,55]. These studies generally concluded that T-DM1 works more efficiently in high-HER2 expressing tumours [28]. However, there is enough evidence to support that TDM-1 loses efficacy in HER2 intratumorally heterogenous cancers [56]. The absence of a by-stander effect of TDM-1 may be a plausible explanation. In this scope, novel anti-HER2 therapies such as trastuzumab and deruxtecan are being developed to overcome this heterogeneity [57].

HER2+/HR+ EBC is a distinct entity associated with different molecular and clinical features compared to HER2+/HR- EBC. The WSG TP phase II-trial compared 12 weeks of T-DM1 with/without endocrine therapy with trastuzumab plus endocrine neoadjuvant therapy in this subgroup. Tumour immunogenicity was associated with better outcome after de-escalated therapy. Luminal A subtype-patients (55%) had improved outcomes despite their rather low pCR rate of 25%. Unfortunately, poor outcomes associated with PIK3CAmut could not be overcome by T-DM1. The authors suggested that further treatment de-escalation strategies should focus on pCR and outcome in Luminal A subtype as HER2+/HR+ tumours are driven by HER2 and ER [58].

Returning to the role of molecular subtypes according to PAM50, a tool to de-escalate therapies, the APT trial also provided important findings. This trial was designed specifically to address the efficacy of adjuvant paclitaxel and trastuzumab in small, node negative HER2+ BC patients. With a 7-year follow up, this combination was associated with excellent long-term outcomes. Distribution of PAM50 intrinsic subtypes in small HER2+ tumours was similar to that previously reported for larger tumours. Where most tumours were identified as HER2-E (66%); subtype distribution differed significantly by HR status and a greater percentage of HR-negative tumours were HER2-E and basal-like. Interestingly, the majority (71%) were categorized as high ROR. However, given the low event rate seen in this study, these data may not be applicable to current practice [29]. In order to further improve these clinical results from APT trial, the ATEMPT trial compared adjuvant T-DM1 and paclitaxel plus trastuzumab in patients with stage I HER2+ BC. TDM-1 was associated with excellent 3-year iDFS, but not with fewer clinically relevant toxicity [59]. Further translational results are anticipated.

Finally, other image-based tailoring strategies have been also explored instead of molecular findings. PHERGain was designed to explore whether omitting chemotherapy in PET responsive patients was feasible. Patients achieving PET response after two cycles of pertuzumab-trastuzumab completed a total of eight cycles and went to surgery, entirely avoiding chemotherapy if pCR was achieved. In contrast, non-responders switched to TCHP for six cycles. Eighty percent of the patients in the HP arm were responders and could spare chemotherapy and 40% of them achieved a pCR. Remarkably, after those two cycles of HP, PET non-responding patients had a very low pCR rate (26%), underscoring that these nonresponding tumours are biologically different [60]. Biomarkers are built into this design as well as IDFS.

In other words, synergic date provided by imagen functional image and molecular subtyping could accuracy select the best responders.

### 4.4. Escalating Therapies: Antidrug Conjugates (ADC)

The current standard of postsurgical systemic treatment in patients with HER2+ disease has been the completion of 1 year of HER2-targeted therapy, irrespective of postneoadjuvant pathological findings. However, patients with residual disease have a substantially worse long-term outcome than those with a pCR. KATHERINE trial is one of the first studies to demonstrate the value of escalating therapy regarding residual disease after neoadjuvant chemotherapy with single or dual HER2 blockade. Adjuvant TDM-1 demonstrated an 11% absolute improvement in iDFS when compared to standard adjuvant trastuzumab [22]. Therefore, it was clearly shown that under some circumstances, more is better. The question lays on identifying who really benefits from switching to the adjuvant ADC. Molecular features may accurately solve this issue. The exploratory biomarker analyses from KATHERINE study showed that *PIK3CA* mutation status was neither prognostic nor predictive of T-DM1 benefits. Moreover, no other assessed biomarker was able to predict T-DM1 benefits.

Interestingly, neoadjuvant chemo and trastuzumab-based treatment led to a lower expression of HER2 when analysing changes in HER2 mRNA from baseline to the time of surgery. Of note, patients with high HER2 expression in the residual disease and therefore, resistant to trastuzumab, had a dismal prognosis when given this drug and appear to be rescued when treated with T-DM1. Those patients with low HER2 mRNA levels at the time of surgery showed a better prognosis but also benefited from T-DM1 [61]. In addition, in an exploratory analysis, those tumours that were converted to HER2 negative at the time of surgery appeared to benefit more from T-DM1 [62]. Perhaps analyses of intrinsic subtypes could lead to a better understanding of this finding.

### 4.5. Escalating Therapies: TKIs and Anti-HER2 AcMo

In an attempt to improve the efficacy of adjuvant systemic therapies, novel anti-HER2 drugs are being explored. Pertuzumab (APHINITY) and neratinib (ExTeNET) have yielded interesting results, while data from some other drugs (TDX or immunotherapy) are still anticipated.

APHINITY, a phase III study in HER2+ early BC patients, showed a significant disease-free survival benefit (3 years) after the addition of pertuzumab to the standard adjuvant trastuzumab plus chemotherapy [63]. Biomarker analyses according to the blueprint signature (BP) of this trial were performed. BP is an 80-gene molecular subtyping test that classifies early BC into functional basal, luminal and HER2 BP-subtypes according to the gene expression signature [64]. A greater benefit of the addition of pertuzumab was suggested in the HER2 BP subtype compared with the other groups. However, further research is warranted to confirm these findings [65].

One year of adjuvant therapy with neratinib has also demonstrated benefits such as extended adjuvant therapy in HER2+ BC, especially in ER+ tumours after completion of 1 year of trastuzumab [66]. To our knowledge, no molecular subtype analyses have been performed. Conversely, in another setting, neratinib demonstrated the benefits for non-overexpressed but HER2 mutated BC [67]. An adaptive trial of neoadjuvant therapy for high-risk HER2 BC patients suggested that neratinib was highly likely to result in higher rates of pCR when added to standard chemotherapy with trastuzumab among patients with HER2+/ER- BC. Patients with a higher risk score according to 70 genes assay appeared to achieve higher benefits from neratinib. In contrast to the Extenet trial, ER-/HER2+ BC patients appeared to achieve the highest benefit [68].

Finally, the NSABP FB-7 trial studied neoadjuvant trastuzumab and/or neratinib combined with chemotherapy. The pCR rate was numerically higher in the double anti-HER2 arm compared to single targeted therapies. The authors reported that an 8-gene signature (using RNA-seq data) correlated with pCR across all arms although this conclusion need to be further validated in larger prospective neoadjuvant trials. An analysis of intrinsic subtypes from whole transcriptome RNA-seq analyses showed the highest pCR rates in the basal and HER2-E subtypes (60% and 61%, respectively) and the lowest in the luminal subtypes (33%). Interestingly, the comparison of intrinsic subtypes between pre and posttreatment tumours revealed that 55% of tumours converted to a normal-like subtype in accordance with data from the PAMELA trial [69].

## 5. Ongoing Trials Based on Molecular Subtyping

Ever since the first anti-HER2 therapy was approved in 1998, eight anti-HER2 drugs have been incorporated into the armamentarium and a good handful of novel compounds are currently being tested. Molecular profiling may guide next generation clinical trials and should be specifically designed in each intrinsic subtype for optimum individualized therapeutic approaches (Table 2).

Risk adapted strategies regarding pCR, similar to the KATHERINE trial design, are necessary. Decrescendo is a phase II de-escalation study evaluating neoadjuvant paclitaxel or docetaxel combined with pertuzumab and trastuzumab. Subjects receiving pCR are planned to complete adjuvant HER2 double blockade, while those with residual invasive disease will escalate to T-DM1.

Efforts must be focused on identifying genomic predictors of pCR to guide treatment de-escalation in the neoadjuvant setting, not only regarding drugs, but also the extent of surgery. ELPIS trial plans to enrol stage I HER2-E BC patients to evaluate if surgery might be omitted if a complete response is achieved following neoadjuvant paclitaxel, trastuzumab, and pertuzumab. Perhaps, these kinds of trials may be the beginning to overcome surgical procedures similar to those in other haemato-oncology disorders.

HER2 negative BC is also a heterogeneous disease and HER2-E subtype can also be identified. PAMILIA phase II study aims to determine whether the addition of HER2-targeted treatment increases the pathologic remission rate in HER2 negative (IHC1+ or 2+ (FISH/SISH-) but HER2-E BC according to PAM50. This study endorses the concept that IHC is not a reliable surrogate of genomic intrinsic subtypes, and that current IHC-based classification may imply a loss of opportunity.

In particular, the HER2-E subtype represents approximately 6.6–11.0% of HR+/HER2- tumours; thus, the incorporation of novel drugs in combination with endocrine therapy can improve patient outcomes, especially in HER2-E subtype. With this rationale, the NEREA trial evaluates targeting EGFR/ERBB2 with neratinib in this group of patients in a metastatic setting.

Similarly, the main hypothesis of the ARIANNA study is that enzalutamide induces a significant proliferative arrest in HR+/HER2-negative BC falling into the PAM50 HER2-E subtype.

As HER2 positive BC is often correlated with a high expression of TILs and PD/PD-L1, immunogenic therapeutic strategies seem to be very promising. Therefore, a de-escalating chemotherapy-free phase II trial with a single arm containing dual anti-HER2 blockade with trastuzumab and pertuzumab and the checkpoint inhibitor pembrolizumab is able to evaluate the pCR rate in patients with HER-E BC. Further translational research will be added to gain further insight into the tumour response or resistance to this treatment approach.

Conversely, durvalumab is a drug that also enhances immune system activity. Thus, safety and effectiveness in terms of pCR of this anti-PDL-1, together with trastuzumab and pertuzumab treatment is being evaluated in HER2-E BC patients in the phase II trial DPT.

To conclude, in order to evaluate whether dual blockade promotes changes to biomarkers associated with immunomodulation, a phase II study is being performed on HER2+ advanced BC patients treated with at least 2 prior lines of anti-HER2-targeted therapies.

## 6. Discussion

HER2-positive BC is a biologically heterogeneous disease with different treatment sensitivities and survival outcomes. Although most patients belong to the HER2-E subtype, all four intrinsic molecular subtypes can be identified in this population. PAM50 has been developed as the most accurate genomic assay to define the HER2-E intrinsic subtype. As IHC-HER2 status is not a perfect predictive marker, molecular profiling may accurately define those HER2 addictive tumours and provide deeper insights into potential therapeutic approaches. However, to better portray “HER2 addiction”, other variables may be needed. A platform that includes molecular features and the immune milieu is necessary. Whether such a molecular classifier combined with conventional clinicopathological characteristics will help us to further refine patient stratification remains to be explored in prospective clinical research.

Conventional clinical trials have been designed regardless of these molecular features. Thus, IHC-based classification may lead to inappropriate treatment, but molecular profiling enables personalized treatment approaches.

There is enough evidence to support that there is a multiparametric accurately defined HER2 addicted BC, that derives the most benefit from HER2 blockade and, therefore, remains the best subtype for de-escalating strategies. The question is whether these patients might be cured with dual HER2 blockade while sparing treatment with chemotherapy. However, this appealing de-escalating approach remains exploratory, as the achieved pCR rate is below what salvage HER2 plus chemo regimens provide and, therefore, this hypothesis needs further validation.

Alternately, given the extreme sensitivity of HER2-E tumours to anti-HER2 therapies, single HER2 blockade combined with taxane based chemotherapy could be enough to treat these patients. Thus, double blockade would not always be necessary.

On the other hand, risk-adapted novel trial designs tailoring treatment by delivering novel drugs to treat high risk patients are needed.

## 7. Conclusions

To conclude, each HER2-positive patient needs to be treated individually, in accordance with the biological and clinical characteristics of the tumour and the patient’s own personal conditions and comorbidities. Decision making must balance the risk of disease recurrence, life-threatening or irreversible toxicity risk, and associated costs.

## Figures and Tables

**Table 1:** pCR rate in HER2-E subtype according to PAM50 in single/dual anti-HER2 trials. HT: Hormonotherapy; NA: Not applicable. * Data from TBCRC006/023 Cohort.

Trial	Phase	Setting	Treatment	n	pCR Rate	%HER2-E	pCR Rate in HER2-E	pCR Rate in Other Subtypes
CALGB40601 (Alliance) [11]	III	Neoadjuvant	Paclitaxel plus trastuzumab with or without lapatinib	305	46 vs. 56% (*p* = 0.13) (ypT0/is)	31% (mRNAseq)	70%	34–36% (*p* < 0.001)
PAMELA [23]	II	Neoadjuvant	Trastuzumab plus lapatinib (plus HT if hormone receptor positive) (1)	151	30% (ypT0/is)	67%	41%	10% (*p* = 0.0004)
TBCRC 006 [24]	II	Neoadjuvant	Trastuzumab plus lapatinib (plus HT if hormone receptor positive) (1)	66	27% (ypT0/is)	NA	NA	NA
TBCRC 023 [25]	II	Neoadjuvant	Trastuzumab plus lapatinib (plus HT if hormone receptor positive) 12 weeks or 24 weeks (1)	97	12 weeks: 12% (ypT0/is) 24 weeks: 28% (ypT0/is)	NA	NA	NA
Combined neoadjuvant HER2-positive dataset [26]	II-III included	Neoadjuvant/advanced	Lapatinib or pertuzumab plus trastuzumab	305 (265 samples)	26.4% (ypT0/is)	65.6%	27.4% *	9.8% (*p* = 0.03)
**Trial**	**Phase**	**Setting**	**Treatment**	**n**	**pCR Rate**	**%HER2-E**	**pCR Rate** **in HER2-E**	**pCR Rate** **in Other Subtypes**
Combined analysis of CALGB 40601 (Alliance) and PAMELA clinical [13]	II-III included	Neoadjuvant	Trastuzumab plus lapatinib (plus HT if hormone receptor positive) (1) CALGB 40601 (Alliance) paclitaxel plus trastuzumab with or without lapatinib (PAMELA clinical)	407 samples	NA	62%	48.6%	20.7% (*p* < 0.001)
perELISA [27]	II	Neoadjuvant	Letrozole plus trastuzumab plus pertuzumab	64 (44 molecular responders)	20% (ypT0/is, ypN0)	41%	45%	13.8% (*p* = 0.042)
KRISTINE [28]	III	Neoadjuvant	Trastuzumab plus pertuzumab plus docetaxel plus carboplatin (TCHP) vs. TDM-1 plus Pertuzumab (TDM-1+ P)	444 (354 samples)	55.7% vs. 44.5% (*p* = 0.016) (ypT0/is, ypN0)	54.8%	72.1% vs. 62.2%	32.8% vs. 26.9%
APT [29]	II	Adjuvant	Paclitaxel plus trastuzumab	406	NA	66%	NA	NA

**Table 2:** Molecular subtyping decision-guided ongoing clinical trials in BC.

NCT Clinical Trials.gov:	Trial	Molecular Subtype
NCT04675827	De-escalation Adjuvant Chemo in HER2+/ER-/Node-neg Early BC Patients Who Achieved pCR After Neoadjuvant Chemo & Dual HER2 Blockade (Decrescendo).	NA
NCT04578106	Omission of Surgery in Clinically Low-risk HER2 positive Breast Cancer with High HER2 Addiction and a Complete Response Following Standard Anti-HER2-based Neoadjuvant Therapy (ELPIS).	HER2-E
NCT04817540	Phase II Trial of Anti-HER2 Treatment in HER2-enriched Early Breast Cancer Identified by PAM50 (HER2E-PAM, PAMILIA Study).	HER2-E
NCT04460430	Targeting EGFR/ERBB2 with Neratinib in Hormone Receptor (HR)-Positive/HER2-negative HER2-enriched Advanced/Metastatic Breast Cancer (NEREA).	HER2-E
NCT04142060	Targeting PAM50 Her2-Enriched Phenotype with Enzalutamide in Hormone Receptor Positive/Her2-Negative Metastatic Breast Cancer (ARIANNA).	HER2-E
NCT03988036	A Study with Pembrolizumab in Combination with Dual Anti-HER2 Blockade with Trastuzumab and Pertuzumab in Early Breast Cancer Patients with Molecular HER2-enriched Intrinsic Subtype (Keyriched-1).	HER2-E
NCT03820141	Durvalumab with Trastuzumab and Pertuzumab in HER2-Enriched Breast Cancer (DTP).	HER2-E
NCT02213042	Evaluation of Biomarkers Associated with Response to Subsequent Therapies in Subjects with HER2-Positive Metastatic Breast Cancer.	HER2
